# Population‐wide differentials in HIV service access and outcomes in the Western Cape for men as compared to women, South Africa: 2008 to 2018: a cohort analysis

**DOI:** 10.1002/jia2.25530

**Published:** 2020-06-26

**Authors:** Meg Osler, Morna Cornell, Nathan Ford, Katherine Hilderbrand, Eric Goemaere, Andrew Boulle

**Affiliations:** ^1^ Centre for Infectious Disease Epidemiology and Research School of Public Health and Family Medicine University of Cape Town Cape Town South Africa; ^2^ HIV/AIDS Department and Global Hepatitis Programme World Health Organization Geneva Switzerland; ^3^ Médecins Sans Frontières Southern African Medical Unit Cape Town South Africa; ^4^ Department of Health Provincial Government of the Western Cape Cape Town South Africa; ^5^ Wellcome Centre for Infectious Diseases Research in Africa Institute of Infectious Disease and Molecular Medicine University of Cape Town Cape Town South Africa

**Keywords:** HIV/AIDS, gender, access, mortality, antiretroviral therapy, South Africa

## Abstract

**Introduction:**

Few studies have systematically described population‐level differences comparing men and women across the continuum of routine HIV care. This study quantifies differentials in HIV care, treatment and mortality outcomes for men and women over time in South Africa.

**Methods:**

We analysed population‐wide linked anonymized data, including vital registration linkage, for the Western Cape Province, from the time of first CD4 count. Three antiretroviral therapy guideline eligibility periods were defined: 1 January 2008 to 31 July 2011 (CD4 cell count <200 cells/µL), 1 August 2011 to 31 December 2014 (<350 cells/µL), 1 January 2015 to 31 August 2016 (<500 cells/µL). We estimated care uptake based on service attendance, and modelled associations for men and women with ART initiation and overall, pre‐ART and ART mortality. Separate Cox proportional hazard models were built for each outcome and eligibility period, adjusted for tuberculosis, pregnancy, CD4 count and age.

**Results:**

Adult men made up 49% of the population and constituted 37% of those living with HIV. In 2009, 46% of men living with HIV attended health services, rising to 67% by 2015 compared to 54% and 77% of women respectively. Men contributed <35% of all CD4 cell counts over 10 years and presented with more advanced disease (39% of all first presentation CD4 cell counts from men were <200 cells/µL compared to 25% in women). ART access was lower in men compared to women (AHR 0.79 (0.77 to 0.80) summarized for Period 2) over the entire study). Mortality was greater in men irrespective of ART (AHR 1.08 (1.01 to 1.16) Period 3) and after ART start (AHR 1.15 (1.05 to 1.20) Period 3) with mortality differences decreasing over time.

**Conclusions:**

Compared to women, men presented with more advanced disease, were less likely to attend health care services annually, were less likely to initiate ART and had higher mortality overall and while receiving ART care. People living with HIV were more likely to initiate ART if they had acute reasons to access healthcare beyond HIV, such as being pregnant or being co‐infected with tuberculosis. Our findings point to missed opportunities for improving access to and outcomes from interventions for men along the entire HIV cascade.

## 1 INTRODUCTION

South Africa (SA) has the largest antiretroviral therapy (ART) programme in the world [1, 2]. Coverage of those eligible for ART is improving year‐on‐year [3] but men living with HIV continue to have inferior access to HIV care and treatment outcomes compared to women[4, 5]. Improvements in mortality on ART are slowing, losses to care happen at all levels of the HIV cascade and a large number of patients, predominantly men, continue to present with advanced HIV disease in spite of the widespread access to ART [6, 7]. Identifying patients who are less likely to test, present to care, start ART and remain on effective ART, provides opportunities to adjust service delivery models to be more responsive to the needs of these patients.

A range of studies have shown that HIV‐infected men in South Africa are less likely to access care than women, and present with more advanced disease [1, 4, 8]. Across the region, men on ART also have higher mortality than women after initiating antiretroviral drugs [5, 9]. Any exploration of differential access to care requires consideration of the changing eligibility criteria for ART access. While patient cohorts and randomized trials have demonstrated reduced morbidity and mortality risks in patients accessing ART at higher CD4 cell counts [10], there is limited real‐world data from high HIV‐burden countries exploring the impact of changing treatment guidelines on differential outcomes by sex.

The aim of this analysis is to describe in the Western Cape Province of South Africa, population‐wide differences of men compared to women in presentation with HIV, access to ART and mortality on ART around the successive CD4 cell count eligibility thresholds as guidelines evolved.

## 2 METHODS

### 2.1 Setting and data sources

The Western Cape is one of nine provinces in South Africa, with a population of 6.7 million, an estimated 439,136 people living with HIV and 260,734 people on ART in 2018 [11]. The vast majority of people living with HIV seek care in the public sector. ART was first available in pilot projects as of 2001, and coverage accelerated after 2004 when ART provision became national policy [12]. CD4 cell count monitoring of all HIV positive patients has been provided since programme inception. In March 2013, CD4 cell count monitoring after one year on ART among virologically suppressed and clinically‐well patients with CD4 ≥200 cells/µL was no longer recommended.

Full details of the ART programme evolution are described elsewhere [12, 13, 14]. From 2004 to March 2010, an adult was eligible for ART with a CD4 cell count <200 cells/µL or a WHO stage IV illness. In April 2010, national guideline revisions increased ART eligibility to include CD4 cell counts of 350 cells/µL for pregnant women and those with active tuberculosis (TB) [15]. In August 2011, eligibility was expanded to include all patients with a CD4 cell count <350 cells/µL [16]. In April 2013, all TB patients and pregnant women were eligible regardless of their CD4 cell count [17]. In January 2015, guidelines were changed again to expand eligibility to all patients with a CD4 < 500 cells/µL [18]. Finally, in September 2016, universal access was introduced, and all HIV infected people were eligible to start ART regardless of CD4 cell count as per WHO guidelines [2, 19]. These changes for patients without other health conditions conferring ART eligibility are summarized into three CD4 cell count ART‐eligibility eras in Tables 1 and 2 and referenced throughout this paper, period 1: 1 January 2008 to 31 July 2011 (CD4 cell count <200 cells/µL), period 2: 1 August 2011 to 31 December 2014 (CD4 cell count <350 cells/µL), period 3: 1 January 2015 to 31 August 2016 (<500 cells/µL).

**Table 1:** HIV status and CD4 cell count results for the Western Cape population and the study population

CD4 count threshold time period	Western cape population							Study population				
	Men, N (%)		Women, N (%)		Total	Men, %	Women, %	Men, N (%)		Women, N (%)		Total
Incident cases^a^						Incidence (/100 py)^a^						
2009	9,219	(39)	14,180	(61)	23,399	0.48	0.71					
2012	8,491	(40)	12,882	(60)	21,373	0.42	0.62					
2015	7,874	(40)	11,826	(60)	19,700	0.37	0.54					
2018	6,457	(40)	9,830	(60)	16,287	0.28	0.43					
Prevalent cases^a^						Prevalence (%)^a^						
2009	103,334	(37)	173,366	(63)	276,700	5.10	7.99					
2012	123,565	(37)	210,192	(63)	333,757	5.73	9.18					
2015	143,662	(37)	246,777	(63)	390,439	6.25	10.11					
2018	161,050	(37)	278,086	(63)	439,136	6.62	10.75					
Unique patient visits/year						% of cases						
2009	47,268	(33)	94,111	(67)	141,379	46	54					
2012	74,889	(33)	148,671	(67)	223,560	61	71					
2015	96,386	(34)	190,914	(66)	287,300	67	77					
First CD4 count, per person												
Period 1	66,351	(34)	130,584	(66)	196,935			30,892	(29)	74,809	(71)	105,701
Period 2	56,664	(38)	93,929	(62)	150,593			31,303	(33)	60,368	(67)	91,671
Period 3	29,084	(39)	45,786	(61)	74,870			15,269	(34)	27,979	(66)	43,248
First CD4 count categories						%in period						
0 to 199												
Period 1	27,962	(44)	35,923	(56)	63,885	42	28	12,645	(40)	19,221	(60)	31,866
Period 2	20,780	(49)	21,235	(51)	42,015	37	23	11,568	(46)	13,408	(54)	24,976
Period 3	10,557	(52)	9,842	(48)	20,399	36	21	5,519	(49)	5,824	(51)	11,343
200 to 349												
Period 1	17,899	(33)	35,949	(67)	53,848	27	28	8,383	(29)	20,573	(71)	28,956
Period 2	15,085	(39)	23,381	(61)	38,466	27	25	8,217	(35)	15,009	(65)	23,226
Period 3	7,804	(41)	11,245	(59)	19,049	27	25	4,059	(37)	6,833	(63)	10,892
350 to 500												
Period 1	10,810	(28)	27,969	(72)	38,779	16	21	5,150	(24)	16,567	(76)	21,717
Period 2	10,373	(33)	21,106	(67)	31,479	18	22	5,692	(29)	13,605	(71)	19,297
Period 3	5,387	(34)	10,560	(66)	15,947	19	23	2,777	(30)	6,483	(70)	9,260
>500												
Period 1	9,680	(24)	30,743	(76)	40,423	15	24	4,714	(20)	18,448	(80)	23,162
Period 2	10,426	(27)	28,207	(73)	38,633	18	30	5,826	(24)	18,346	(76)	24,172
Period 3	5,336	(27)	14,139	(73)	19,475	18	31	2,914	(25)	8,839	(75)	11,753
Tuberculosis at first CD4						%in period						
Period 1	15,445	(53)	13,798	(47)	29,243	23	11	7,170	(50)	7,179	(50)	14,349
Period 2	12,870	(58)	9,261	(42)	22,131	23	10	7,359	(56)	5,802	(44)	13,161
Period 3	6,551	(61)	4,246	(39)	10,797	23	9	3,603	(58)	2,577	(42)	6,180
Pregnancy at first CD4						%in period						
Period 1			28,258	(100)	28,258		22			20,764	(100)	20,764
Period 2			17,348	(100)	17,348		18			13,335	(100)	13,335
Period 3			9,499	(100)	9,499		21			6,880	(100)	6,880
New ART enrolment												
Period 1	37,381	(32)	80,806	(68)	118,187			22,061	(28)	57,857	(72)	79,918
Period 2	36,738	(35)	67,089	(65)	103,827			22,743	(32)	47,974	(68)	70,717
Period 3	19,506	(36)	34,551	(64)	54,057			10,847	(33)	22,277	(67)	33,124
Deaths												
Period 1	12,951	(46)	15,346	(54)	28,297			8,895	(44)	11,468	(56)	20,363
Period 2	8,108	(52)	7,631	(48)	15,739			6,103	(50)	6,114	(50)	12,217
Period 3	2,646	(53)	2,300	(47)	4,946			1,888	(52)	1,756	(48)	3,644

^a^ Thembisa Model outputs v4.2 for all > 14 years old (estimates for June each year) [28],(ART) antiretroviral therapy;(py) person years ; (cs) cases; (Period 1) 1 January 2008 to 31 July 2011 [CD4 count eligibility <200 cells/µL]; (Period 2) 1 August 2011 to 31 December 2014 (CD4 count eligibility <350 cells/µL); (Period 3) 1 January 2015 to 31 August 2016 (CD4 count eligibility threshold <500 cells/µL). Please note: Absolute numbers are divided by the number of first CD4 count presentations in the same year for tuberculosis, pregnancy and CD4 count indicators.

**Table 2:** Mortality for men, baseline characteristics and crude and adjusted hazard ratios as compared to non‐pregnant women

Survival analysis	Threshold time period	Adults N (% men)		Median CD4 (M:W)	Median Age (M:W)	Started ART N (% men)		Men, crude HR (95% CI)	Men, AHR (95% CI)
Starting ART	Period 1	104,472	(29)	247:332	35:29	47,907	(31)	1.03 (1.01, 1.05)	0.78 (0.76, 0.79)
	Period 2	87,131	(35)	273:368	35:29	55,758	(34)	0.98 (0.96, 0.99)	0.79 (0.77, 0.80)
	Period 3	38,465	(39)	278:378	35:29	28,235	(37)	0.94 (0.91, 0.96)	0.80 (0.78, 0.82)

Survival analysis	Threshold time period	Adults N (% men)		Median CD4 (M:W)	Median Age (M:W)	Deaths N (% men)		Men, crude HR (95% CI)	Men, AHR (95% CI)
Pre‐ART mortality	Period 1	104,472	(29)	247:332	35:29	8,914	(45)	1.71 (1.63, 1.78)	1.12 (1.07, 1.17)
	Period 2	87,131	(35)	273:368	35:29	4,919	(50)	1.47 (1.39, 1.56)	1.03 (0.97, 1.09)
	Period 3	38,465	(39)	278:378	35:29	1,532	(51)	1.24 (1.12, 1.38)	0.92 (0.83, 1.02)
ART mortality	Period 1	79,918	(28)	227:313	35:29	3,651	(45)	1.85 (1.73, 1.98)	1.17 (1.09, 1.25)
	Period 2	70,717	(32)	242:344	36:29	4,243	(50)	1.83 (1.72, 1.95)	1.14 (1.07, 1.21)
	Period 3	33,124	(33)	246:359	36:29	2,020	(52)	1.85 (1.69, 2.02)	1.15 (1.05, 1.26)
Mortality, irrespective of ART	Period 1	105,701	(29)	247:332	35:29	10,134	(46)	1.75 (1.68, 1.82)	1.17 (1.13, 1.23)
	Period 2	91,671	(34)	273:368	35:29	8,325	(50)	1.70 (1.63, 1.78)	1.13 (1.08, 1.18)
	Period 3	43,248	(35)	278:378	35:29	3,536	(52)	1.66 (1.55, 1.78)	1.08 (1.01, 1.16)

(Period 1) 1 January 2008 to 31 July 2011 (CD4 cell count eligibility <200 cells/µL); (Period 2) 1 August 2011 to 31 December 2014 (CD4 cell count eligibility <350 cells/µL); (Period 3) 1 January 2015 to 31 August 2016 (CD4 cell count eligibility threshold <500 cells/µL). Adjusted for baseline CD4 cell count and age categories, tuberculosis and pregnancy at first CD4 test. The analyses covered 3 years from first CD4 cell count or ART initiation (for ART mortality only). AHR, Adjusted hazard ratio; ART, antiretroviral therapy; CI, confidence interval; HR, Hazard Ratio; M:W, men vs. women; CD4, CD4 cell count in cells/µL; (N) number of people.

All public‐sector laboratory testing was done by the National Health Laboratory Service and province‐wide digitized results were available from 2007 onwards. The province has successfully established a patient registration system which shares a unique health identifier and Patient Master Index (PMI) across both hospital and ambulatory services [20]. This has facilitated the linkage of data from hospital, laboratory and pharmacy sources, as well as electronic disease registers such as those for HIV and tuberculosis [3]. Information on deaths was extracted from the National Population Register (NPR) [21], which classified deaths as either natural or unnatural. The process of linking all data to the PMI is formalized through the Provincial Health Data Centre [22]. All data provided for analyses are pre‐anonymized but linkable based on a privacy‐preserving random key.

The study was approved by the Western Cape Department of Health and the University of Cape Town Human Research Ethics Committee. A waiver of consent was issued by the committee due to the use of anonymized data.

### 2.2 Study population

**Figure 1:**
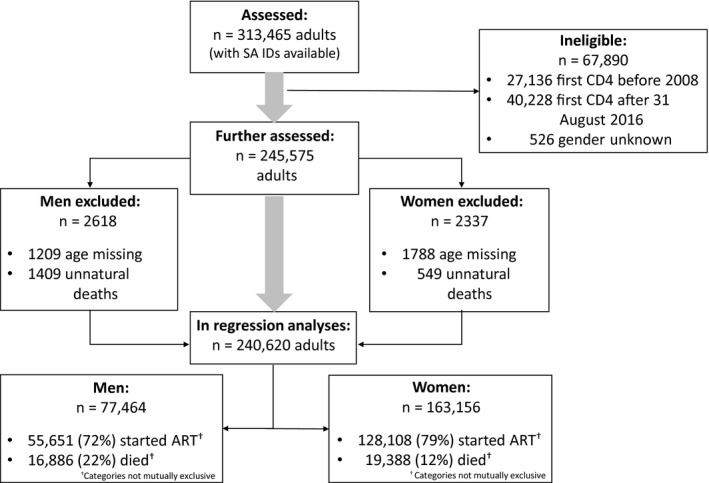
Patient flow chart describing the cohort of patients included in the survival analyses.

The study population consisted of all patients seeking HIV treatment and care in the Western Cape public‐sector health services with their first pre‐ART CD4 cell count between 1 January 2007 and 31 August 2016. We followed up the study population for three years from their first CD4 cell count, with the last follow‐up data in 2018. We excluded children and adolescents less than 16 years of age on the date of their pre‐ART CD4 cell count, people without a CD4 test result prior to the ART start date, any record with a first CD4 specimen taken at hospital, and anyone recorded as having an unnatural death. We intentionally excluded patients tested in hospital to focus on patients with ambulatory‐care CD4 cell counts who would be evaluated for ART eligibility based on CD4 cell count rather than co‐morbidities Figure 1.

### 2.3 Study design and key variables

The study comprises a cohort survival analysis by sex of time to ART and time to death from natural causes. The survival analyses were restricted to patients with a South African identification (SA ID) number (57% of the total cohort), a requirement for linkage with data from the NPR [21]. A separate model was fitted for each eligibility time period and outcome.

HIV testing is often done outside healthcare facilities using point of care tests which do not get recorded into routine digital health records, therefore the first CD4 cell count for each patient was defined in this study as the first presentation to HIV care services. We have defined the proportion of patients utilising HIV services as the number of unique patients having a recorded encounter at health care facilities (lab test, visit data, medication pick up) divided by the number of prevalent cases for the same annual time period (2009, 2012, 2015, 2018), stratified by sex. Data from the NPR distinguish natural from non‐natural causes. Mortality was defined as death from natural causes. Survival analyses were restricted to three years after first presentation with HIV.

We included sex, age, first CD4 cell count, tuberculosis (TB) and pregnancy as baseline variables in adjusted models for the survival analyses of time to ART and time to death after presentation or ART initiation. The data in the variable sex were split into three categories, men, non‐pregnant women and pregnant women. Both CD4 cell counts (0 to 49, 50 to 99, 100 to 199, 200 to 349, 350 to 499, ≥500 cells/µL) and age (16 to 24, 25 to 34, 35 to 44, 45+) were analysed in the described categories. We added one day to death dates if the patient was recorded as dying on the date of entry into the analyses.

### 2.4 Statistical analysis

The baseline characteristics of people living with HIV and with a SA ID recorded were described by sex with summary statistics (absolute number, proportions, medians and interquartile ranges (IQR)).

Time to ART and time to death were analysed from the date of the first CD4 cell count. Time to death for those on ART was analysed from their ART initiation date, whereas the baseline CD4 count was at initial presentation and not necessarily ART initiation. We used Cox proportional hazards to assess crude and adjusted associations between baseline characteristics and mortality. Results are presented as adjusted hazard ratios with 95% confidence intervals. The proportional hazards assumption was tested using graphs and Schoenfeld residuals for sex, TB and pregnancy.

Data were analysed using STATA 14.2 (STATA Corporation).

## 3 RESULTS

Men 15 years of age and older comprised 49% the population and constituted 37% of the population living with HIV in the Western Cape from 2008 through 2018 Table 1. In the total cohort, men were less likely than women to be newly diagnosed with HIV by the health services Table 1, although more recently the sex distribution of first‐ever CD4 cell counts has tracked population prevalence more closely. Men were less likely (8% or more) to utilize health care services annually, with 46% of men estimated to be living with HIV recorded as attending health care in 2009, rising to 67% in 2015 (in comparison to 54% to 77% in women respectively). Men presented with more advanced HIV disease than women (39% of first‐ever CD4 cell counts in men were <200 cells/µL over the entire study period in comparison to 25% in woman).

Men living with HIV and first accessing care were more than twice as likely as women to be co‐infected with TB throughout the entire study period (23% of men vs. 10% of women) Table 1. The co‐infection proportion remained at 23% throughout each period for men but decreased slightly over time among women presenting to HIV care. The number of women pregnant at first CD4 test was about double that of women co‐infected with TB in each period. Across all three eligibility eras, in the general and study populations, men were less likely to start ART than women. Overall, 62% of men and 67% of women who presented for their first CD4 test during the study started ART within three years of their first CD4 test.

Men were less likely to start ART and more likely to die, regardless of whether they started ART, throughout the study Table 2, when compared to non‐pregnant women. Illustratively, after adjusting for age, CD4 cell count, tuberculosis and pregnancy at baseline, men were 22% (adjusted hazard ratio [AHR] 0.78, 95% CI 0.76 to 0.79) less likely to start ART in comparison to woman during Period 1.Men had higher mortality overall (AHR 1.17, 95% CI 1.09 to 1.25 in the 200 cell/µL threshold), higher mortality in the first eligibility period prior to initiating ART (AHR 1.12, 95% CI 1.07 to 1.17) and higher mortality on ART (AHR 1.17, 95% CI 1.09 to 1.25 in the first era). Mortality pre‐ART and mortality overall appear to be decreasing over time in comparison to women, narrowing the mortality difference.

Access to ART by sex was confounded by pregnancy, initial CD4 cell count and TB, with a proportion of women presenting pregnant, and men more likely to present with advanced disease or tuberculosis, each independently associated with ART access Tables 2 and 3a. When considering crude hazard ratios, men, non‐pregnant and pregnant women all had similar uptake of ART services. However, the adjusted hazard ratios in Period 2 for example, show that men who were not co‐infected with tuberculosis had the lowest uptake, 21% less than non‐pregnant women. TB and pregnancy remained independently associated with starting ART (34% and 17% more likely to initiative ART respectively), while there was a consistent inverse association after adjustment between CD4 count and starting ART Table 3.

**Table 3:** Cox proportional hazard models for associations with starting ART and with mortality in HIV and ART care

	Period 1				Period 2				Period 3			
	HR	95% CI	AHR	95% CI	HR	95% CI	AHR	95% CI	HR	95% CI	AHR	95% CI
a. Starting ART												
Sex												
Women	1	ref	1	ref	1	ref	1	ref	1	ref	1	ref
Men	1.03	(1.01, 1.05)	0.78	(0.76, 0.79)	0.98	(0.96, 0.99)	0.79	(0.77, 0.80)	0.9	(0.91, 0.96)	0.80	(0.78 to 0.82)
Pregnant	0.62	(0.60, 0.64)	0.82	(0.80, 0.84)	1.00	(0.97, 1.02)	1.17	(1.14, 1.20)	1.2	(1.20, 1.29)	1.36	(1.31, 1.41)
Tuberculosis	2.05	(2.00, 2.10)	1.16	(1.14, 1.19)	1.96	(1.92, 2.01)	1.34	(1.31, 1.37)	1.61	(1.56, 1.67)	1.36	(1.32, 1.41)
Age												
15 to 24	1	ref	1	ref	1	ref	1	ref	1	ref	1	ref
25 to 34	1.48	(1.44, 1.52)	1.20	(1.17, 1.23)	1.13	(1.11, 1.16)	1.01	(0.99, 1.04)	1.06	(1.02, 1.09)	1.00	(0.97, 1.03)
35 to 44	1.84	(1.79, 1.89)	1.33	(1.29, 1.37)	1.27	(1.24, 1.30)	1.08	(1.04, 1.10)	1.15	(1.11, 1.29)	1.07	(1.03, 1.11)
≥45	1.80	(1.74, 1.86)	1.25	(1.21, 1.30)	1.20	(1.16, 1.23)	1.02	(0.99, 1.06)	1.12	(1.08, 1.17)	1.07	(1.03, 1.11)
First CD4												
0 to 49	1	ref	1	ref	1	ref	1	ref	1	ref	1	ref
50 to 99	1.07	(1.03, 1.11)	1.09	(1.05, 1.13)	1.08	(1.03, 1.12)	1.10	(1.06, 1.15)	1.07	(1.00, 1.14)	1.11	(1.04, 1.18)
100 to 199	0.94	(0.91, 0.98)	1.00	(0.96, 1.03)	0.90	(0.87, 0.94)	0.96	(0.93, 1.00)	0.98	(0.93, 1.04)	1.07	(1.02, 1.13)
200 to 349	0.46	(0.45, 0.48)	0.50	(0.48, 1.03)	0.75	(0.72, 0.77)	0.81	(0.78, 0.84)	0.87	(0.83, 0.92)	0.97	(0.92, 1.02)
350 to 499	0.20	(0.19, 0.21)	0.22	(0.21, 0.23)	0.36	(0.35, 0.38)	0.39	(0.38, 0.41)	0.79	(0.75, 0.83)	0.87	(0.82, 0.92)
≥500	0.09	(0.08, 0.09)	0.10	(0.09, 0.10)	0.23	(0.22, 0.24)	0.25	(0.24, 0.26)	0.40	(0.38, 0.42)	0.43	(0.41, 0.46)
b. Pre‐ART Mortality												
sex												
Women	1	ref	1	ref	1	ref	1	ref	1	ref	1	ref
Men	1.71	(1.63, 1.78)	1.12	(1.07, 1.17)	1.47	(1.39, 1.56)	1.03	(0.97, 1.09)	1.24	(1.12, 1.38)	0.92	(0.83, 1.02)
Pregnant	0.43	(0.40, 0.47)	0.70	(0.65, 0.75)	0.34	(0.92, 0.39)	0.60	(0.52, 0.69)	0.41	(0.30, 0.54)	0.70	(0.52, 0.94)
Tuberculosis	3.40	(3.24, 356)	1.43	(1.36, 1.51)	4.25	(4.00, 4.53)	1.55	(1.44, 1.66)	4.83	(4.35, 5.37)	1.86	(1.66, 2.10)
Age												
15 to 24	1	ref	1	ref	1	ref	1	ref	1	ref	1	ref
25 to 34	1.96	(1.82, 2.10)	1.50	(1.39, 1.61)	2.08	(1.85, 2.32)	1.61	(1.43, 1.80)	2.57	(2.03, 3.25)	1.91	(2.51, 2.42)
35 to 44	3.60	(3.35, 3.88)	2.19	(2.02, 2.36)	3.84	(3.43, 4.30)	2.43	(2.16, 2.73)	4.91	(3.89, 6.20)	3.00	(2.37, 3.80)
≥45	6.60	(6.12, 7.11)	3.49	(3.23, 3.78)	7.49	(6.70, 8.34)	4.26	(3.79, 4.78)	8.97	(7.15, 11.26)	5.03	(3.99, 6.35)
First CD4												
0 to 49	1	ref	1	ref	1	ref	1	ref	1	ref	1	ref
50 to 99	0.79	(0.73, 0.85)	0.85	(0.78, 0.91)	0.84	(0.76, 0.93)	0.90	(0.81, 0.99)	0.75	(064, 0.89)	0.85	(0.72, 1.00)
100 to 199	0.46	(0.43, 0.50)	0.59	(0.55, 0.64)	0.45	(0.41, 0.50)	0.58	(0.54, 0.63)	0.40	(0.34, 0.46)	0.55	(0.47, 0.65)
200 to 349	0.21	(0.20, 0.23)	0.32	(0.30, 0.35)	0.21	(0.19, 0.23)	0.33	(0.30, 0.36)	0.17	(0.15, 0.20)	0.30	(0.25, 0.35)
350 to 499	0.11	(0.10, 0.12)	0.18	(0.17, 0.20)	0.11	(0.10, 0.12)	0.19	(0.17, 0.21)	0.10	(0.86, 0.13)	0.19	(0.15, 0.23)
≥500	0.85	(0.08,0.09)	0.14	(0.13, 0.15)	0.09	(0.08, 0.09)	0.14	(0.13, 0.15)	0.07	(0.06, 0.84)	0.18	(0.11, 0.15)
c. ART mortality												
Sex												
Women	1	ref	1	ref	1	ref	1	ref	1	ref	1	ref
Men	1.85	(1.73, 1.98)	1.17	(1.09, 1.25)	1.83	(1.72, 1.95)	1.14	(1.07, 1.21)	1.85	(1.69, 2.02)	1.15	(1.05, 1.26)
Pregnant	0.41	(0.36, 0.47)	0.74	(0.65, 0.85)	0.40	(0.35, 0.46)	0.69	(0.60, 0.79)	0.29	(0.23, 0.34)	0.49	(0.40, 0.61)
Tuberculosis	0.59	(3.35, 3.84)	1.51	(1.40, 1.62)	3.90	(3.66, 4.14)	1.70	(1.59, 1.82)	4.15	(3.80, 4.54)	1.88	(1.70, 2.08)
Age												
15 to 24	1	ref	1	ref	1	ref	1	ref	1	ref	1	ref
25 to 34	2.03	(1.80, 2.29)	1.36	(1.20, 1.54)	2.00	(1.77, 2.25)	1.36	(1.21, 1.54)	2.48	(2.05, 3.00)	1.79	(1.48, 2.17)
35 to 44	3.74	(3.31, 4.24)	1.96	(1.73, 2.23)	3.77	(3.34, 4.24)	2.00	(1.77, 2.26)	4.61	(3.81, 5.58)	2.44	(2.01, 2.97)
≥45	6.36	(5.60, 7.23)	3.32	(2.90, 3.79)	6.57	(5.83, 7.41)	3.51	(3.10, 3.97)	9.11	(7.56, 10.97)	4.81	(3.97, 5.82)
First CD4												
0 to 49	1	ref	1	ref	1	ref	1	ref	1	ref	1	ref
50 to 99	0.59	(0.54, 0.65)	0.65	((0.58, 0.71)	0.59	(0.54, 0.65)	0.63	(0.57, 0.69)	0.63	(0.55, 0.73)	0.70	(0.60, 0.80)
100 to 199	0.33	(0.30, 0.36)	0.41	(0.38, 0.45)	0.33	(0.30, 0.36)	0.42	(0.39, 0.46)	0.38	(0.34, 0.44)	0.52	(0.46, 0.60)
200 to 349	0.15	(0.13, 0.16)	0.21	(0.19, 0.23)	0.16	(0.14, 0.36)	0.24	(0.22, 0.27)	0.17	(0.15, 0.20)	0.30	(0.26, 0.34)
350 to 499	0.07	(0.06, 0.85)	0.11	(0.10, 0.13)	0.10	(0.09, 0.11)	0.17	(0.15, 0.19)	0.11	(0.09, 0.13)	0.21	(0.18, 0.25)
≥500	0.04	(0.03, 0.05)	0.07	(0.05, 0.08)	0.07	(0.06, 0.08)	0.12	(0.10, 0.14)	0.10	(0.09, 0.12)	0.21	(0.17, 0.25)
d. Overall mortality												
Sex												
Women	1	ref	1	ref	1	ref	1	ref	1	ref	1	ref
Men	1.75	(1.68, 1.82)	1.18	(1.13, 1.23)	1.70	(1.63, 1.78)	1.13	(1.08, 1.18)	1.66	(1.55, 1.78)	1.08	(1.01, 1.16)
Pregnant	0.38	(0.35, 0.41)	0.68	(0..62, 0.73)	0.32	(0.29, 0.35)	0.56	(0.50, 0.62)	0.27	(0.23, 0.33)	0.47	(0.40, 0.56)
Tuberculosis	3.34	(3.20, 3.48)	1.46	(1.39, 1.52)	3.74	(3.58, 3.91)	1.55	(1.47, 1.63)	4.20	(3.92, 4.49)	1.74	(1.61, 1.87)
Age												
15 to 24	1	ref	1	ref	1	ref	1	ref	1	ref	1	ref
25 to 34	2.03	(21.88, 2.18)	1.45	(1.34, 1.56)	2.07	(1.89, 2.56)	1.48	(1.35, 1.62)	2.57	(2.21, 2.98)	1.88	(1.62, 2.19)
35 to 44	3.64	(3.38, 3.92)	2.07	(1.92, 2.24)	3.91	(3.58, 4.27)	2.20	(2.01, 2.41)	4.93	(4.25, 5.71)	2.71	(2.33, 3.16)
≥45	6.96	(6.45, 7.51)	3.91	(3.61, 4.23)	7.81	(7.15, 8.52)	4.38	(4.01, 4.80)	9.98	(8.63, 11.53)	5.47	(4.71, 6.34)
First CD4												
0 to 49	1	ref	1	ref	1	ref	1	ref	1	ref	1	ref
50 to 99	0.56	(0.53, 0.60)	0.61	(0.57, 0.65)	0.59	(0.55, 0.63)	0.62	(0.57, 0.66)	0.58	(0.52, 0.65)	0.06	(0.57, 0.70)
100 to 199	0.30	(0.28, 0.32)	0.37	(0.35, 0.39)	0.31	(0.29, 0.33	0.39	(0.37, 0.42)	0.32	(0.29, 0.35)	0.04	(0.39, 0.47)
200 to 349	0.16	(0.15, 0.17)	0.23	(0.22, 0.25)	0.15	(0.14, 0.16)	0.22	(0.20, 0.24)	0.14	(0.13, 0.16)	0.24	(0.21, 0.27)
350 to 499	0.10	(0.09, 0.11)	0.15	(0.14, 0.16)	0.10	(0.09, 0.11)	0.17	(0.15, 0.18)	0.09	(0.08, 0.10)	0.17	(0.14, 0.19)
≥500	0.07	(0.06, 0.08)	0.11	(0.10, 0.12)	0.09	(0.08, 0.09)	0.14	(0.13, 0.15)	0.09	(0.08, 0.10)	0.16	(0.14, 0.18)

Category; (Period 1) 1 January 2008 to 31 July 2011 [CD4 count eligibility <200 cells/µL]; (Period 2) 1 August 2011 to 31 December 2014 (CD4 count eligibility <350 cells/µL); (Period 3) 1 January 2015 to 31 August 2016 (CD4 count eligibility threshold <500 cells/µL). Sex was split into three mutually exclusive categories, men, women and pregnant women. The analyses covered three years from first CD4 count for a, b and d, and from ART initiation for c. (AHR) Adjusted hazard ratio; ART, antiretroviral therapy; CI, confidence interval; HR, crude Hazard Ratio; ref, reference.

Mortality in people living with HIV was highly confounded by their baseline CD4 cell count, TB, pregnancy in women, and age Tables 2 and 3b,c. Although men in Period 2 for example had higher mortality risk during pre‐ART care compared to non‐pregnant women prior to adjustment, this difference did not persist after adjustment Tables 2 and 3b. Co‐infection with TB was independently associated with a 55% increase in pre‐ART mortality. As shown in Table 1, men were twice as likely to be infected with TB when presenting to HIV care and therefore more likely to die during this interval prior to initiating ART. This association between TB and mortality persisted for all mortality outcomes in all periods. By contrast pregnancy and higher CD4 counts reduced the risk of mortality pre‐ART, on ART and regardless of ART.

## 4 DISCUSSION

Using clinical, laboratory, pharmaceutical and vital registry data linked at patient level for all adults with HIV in the Western Cape, we analysed differences for men and women in the HIV cascade over the past decade. Overall, men were less likely to present for HIV care than women, represented in this analysis by their first recorded CD4 cell count, and more likely to be diagnosed due to concurrent tuberculosis infection. In analyses limited to patients with SA IDs, across all three eligibility eras, men consistently had both lower health care utilization and enrolment on ART and higher mortality across the entire cascade.

### 4.1 Presenting with HIV and accessing HIV care

Late presentation has been cited as one of the main reasons for ongoing HIV‐associated morbidity and for mortality after starting ART, especially in men [5, 6, 9]. Although women and men presented earlier to HIV care over time based on first CD4 cell count values, the trend was more pronounced in women.

Whereas our findings in the earlier periods concur with other studies which have found men less likely to access HIV care in similar settings [23, 24], we observed a narrowing of this differential in later periods of this study, approaching what would be expected based on sex‐specific HIV prevalence estimates. Men continued to present with lower CD4 cell counts and have a larger proportion of TB co‐infection, which seems to be increasing over time. In addition, numerous studies have found that women, via reproductive health services, have more opportunities to test for HIV and therefore present earlier for care and treatment while not otherwise symptomatic [23, 24, 25]. This study’s results validate that finding, showing pregnant women were 38% more likely to access ART in comparison to men not infected with TB. Recently scaled‐up interventions that increase uptake of HIV and TB testing and care by men, such as community‐based mobile testing units, may be contributing to the gains in access to HIV care seen in men in recent eligibility eras of this study [24, 26, 27]. Model results by Johnson et al. in 2019, have also estimated lower HIV testing among men (84.5% vs. 91.2% in women), as well as lower testing (irrespective of sex) in adolescent age groups compared to adults. The MicroCOSM model results suggest home‐based testing coupled with the opportunity of self‐testing will most likely yield the greatest increase of newly diagnosed people infected with HIV by 2030 and increase the fraction of both men and young adults tested [28]. Changes may also be due to a saturation effect being reached in women earlier than in men, due to the higher proportion of women who already know their HIV status [29].

### 4.2 Antiretroviral therapy access

Men continue to have lower rates of ART enrolment after their first eligible CD4 cell count. Across all three time periods, men were 20% to 22% less likely to start antiretroviral drugs and therapy and that estimation held relatively stable regardless of eligibility era. In the adjusted model, the divergence to poorer ART initiation in men was largely driven by TB co‐infection (largely among men) and pregnancy amongst women. Men not co‐infected with tuberculosis were 21% less likely to start ART than non‐pregnant women, and 38% less likely than pregnant women (summarized for Period 2). When comparing men with and without tuberculosis co‐infection, men not co‐infected were 26% less likely to start ART. Pregnancy and TB infection provide a natural entry point to health care as well as HIV testing and treatment. Poorer access for men to treatment has been widely noted [24, 28, 30]. This study provides strong evidence of the need for clinical service interventions oriented to assisting men (specifically those not accessing health services for other reasons) link to ART care after initial presentation.

### 4.3 Mortality after initial CD4 cell count assessment

Although men had increased pre‐ART mortality compared to women in Period 1 of the study, this difference narrowed over time as CD4 cell count criteria widened to include people earlier in HIV disease progression. As men continue to present to HIV services with more advanced HIV disease [6], specific diagnostic interventions for co‐morbidities associated with advanced HIV disease are needed. These include a Lipoarabinomannan (LAM) antigen tests for TB and cryptococcosis (CrAg) antigen tests, which could be guided by immunodeficiency represented by CD4 cell count testing [31]. Alternatively, in countries where routine CD4 cell counts are not done at baseline, interventions could be based on algorithms for advanced disease risk in which sex may play a role.

### 4.4 Mortality after ART initiation

Men continued to have inferior mortality outcomes on ART over the ten years of this study. Similar results have been widely published [5, 8, 30, 32]. In our analyses, deficits in life expectancy appear largely related to HIV and TB [32], with men showing higher rates of TB co‐infection across all eligibility eras. Although similar or greater mortality differentials were observed even in those who were HIV uninfected [5], interventions targeting men, their retention in care, and the co‐morbidities associated with their advanced disease at ART initiation, are warranted.

### 4.5 Interventions to address the access and treatment gaps for men

There have been some successful interventions designed to increase uptake by men carried out in the Western Cape such as the clinics for men [33, 34] in the services run by the local authority, and the after‐hours clinics in Khayelitsha and other areas [35]. These types of services should develop best practice guidelines and be considered for scale‐up where appropriate across South Africa. Nationally and regionally, a range of interventions have been tested to address gaps in the supply and demand side of men’s access to, and uptake of, HIV services [36]. On the supply side, community‐based projects include testing at home and in the workplace, in index patients, partners of pregnant patients, shebeens, sports centres, shopping centres, mobile facilities and through campaigns including the provision of incentives [37], although many of the pilot projects are not scalable, and linkage to care remains challenging. The high uptake of HIV self‐testing within facilities by men suggests a possible universal entry point for testing, given that in Malawi, 80% of those who needed testing had visited a facility within the last two years [38]. On the demand side, most interventions are community‐based, integrated with work on gender equity and reducing gender‐based violence [36], and focus on individual‐level behaviour change. At an international level, UNAIDS launched a report ‘Blind spot: reaching out to men and boys’ and held a regional consultation in May 2019 to workshop a plan to accelerate men’s uptake of HIV services in the region. In July 2019, the IAS convened the first‐ever Men and HIV Forum prior to the 10^th^ IAS Conference on HIV Science, the World Health Organization has established a working group on the issue, and PEPfAR announced the MenStar public‐private collaboration to increase HIV services for men.

### 4.6 Strengths and limitations

The population‐wide linked dataset with mortality data from vital registration is a strength of this study. The study was restricted to those with vital registry linkage (57% of the cohort), but results were similar in patients with and without available civil identifiers, supporting the generalizability of our findings. A further limitation is that in the early eligibility era, it is unclear whether CD4 cell counts were from first‐ever presentation to HIV services. Linked CD4 cell count data were only available from 2007 and some patients would have been in care prior to the first CD4 cell count available for the analysis.

## 5 CONCLUSIONS

Exploring a provincial HIV cascade over ten years, men continued to present with more advanced disease, were less likely to attend HIV care services at least annually, were less likely to initiate ART, were less impacted by guideline provisions and had inferior on‐ART outcomes. Our findings point to missed opportunities for improving access to and outcomes from interventions for men along the entire HIV cascade.

ART services, offered for free in the public sector, have been rapidly scaled‐up in unprecedented magnitude globally, reducing HIV incidence and mortality. However, men have not benefited from these services as much as women due to low uptake. Women and men living with HIV and accessing health care for non‐HIV services are more likely to start ART, with pregnant women 38% more likely and non‐pregnant women 21% more likely to start ART than men not co‐infected with tuberculosis. In addition, men co‐infected with tuberculosis are 5% more likely to start ART than non‐pregnant women and more likely to die while on ART. These results highlight a need to improve ways of enrolling and retaining otherwise healthy people living with HIV in antiretroviral care.

## COMPETING INTEREST

The authors have no competing interests to declare.

## AUTHORS’ CONTRIBUTIONS

MO, AB and MC conceptualized the paper. MO, AB and KH analysed the data. MO, AB, MC, KH, EG and NF wrote the paper. All authors approved the final draft for submission.
